# Cause of Death Predicting Place of Death, by Rurality: Evidence From the Utah Population Database

**DOI:** 10.1093/geroni/igaf122.028

**Published:** 2025-12-31

**Authors:** Attrayee Bandyopadhyay, Rebecca Utz, Caroline Stephens, Michael Hollingshaus

**Affiliations:** University of Utah, Salt Lake City, Utah, United States; University of Utah, Salt Lake City, Utah, United States; University of Utah, Salt Lake City, Utah, United States; University of Utah, Salt Lake City, Utah, United States

## Abstract

Place of death is a quality indicator of end-of-life (EOL) care. How cause of death affects place of death and how place of death varies across rural-urban areas is unexplored. This study uses a cohort drawn from Utah Caregiving Population Study (C-PopS): deaths from natural causes at ages 20+ in Utah between 1998 and 2016 (n = 217,222). The cohort is stratified by rural (n = 42,055), urban (n = 164,296), and frontier (n = 10,871). C-PopS is created through linkage of administrative data, vital statistics, and detailed health records at the individual level. Results show that heart disease, cancer, and other diseases (24.2%, 21.3%, 34.5%) are the top three primary causes of death. Home (37.6%) is most common place of death, followed by hospitals (30.9%) and nursing homes (26.5%). 60.5% of cancer decedents died at home versus 61.2% of dementia decedents in nursing homes. For all other causes, hospital is most common, except for chronic obstructive pulmonary disease (COPD). Home was slightly more common among rural decedents than urban (39.3% vs. 37.3%). A lower percentage of frontier decedents died in nursing homes compared to both rural and urban areas (19.7% vs. 26.9%), indicating lower utilization of nursing homes in frontier areas. This study has important implications in examining how EOL experiences may vary among the residents of rural, urban, and frontier areas of Utah. The findings from this study inform health policies and future interventions to cater to the unmet EOL care needs of the Utah population.

